# Case Report: Bilateral panuveitis with serous ciliary body and choroidal detachment associated with ulcerative colitis

**DOI:** 10.3389/fmed.2026.1750836

**Published:** 2026-05-13

**Authors:** Aimin Sun, Siying Li, Yu Cao, Bohao Wang, Muzi Li, Jinfeng Qu

**Affiliations:** 1 Department of Ophthalmology, Peking University People’s Hospital, Beijing, China; 2 Beijing Key Laboratory of Ocular Disease and Optometry Science, Peking University People’s Hospital, Beijing, China

**Keywords:** choroidal detachment, ciliary body detachment, panuveitis, ulcerative colitis, uveitis

## Abstract

**Purpose:**

To report a rare case of ulcerative colitis (UC)-associated bilateral panuveitis presenting with serous ciliary body detachment and choroidal detachment.

**Case description:**

A 51-year-old Asian woman with a history of UC presented with a 10-day history of bilateral blurred vision, ocular pain, redness, and photophobia. At presentation, her best-corrected visual acuity (BCVA) was 20/60 in the right eye and 20/100 in the left eye, with normal intraocular pressure. Slit-lamp examination showed signs of anterior uveitis. Fundus examination revealed bilateral optic disk edema and peripheral choroidal detachment in the left eye. Indocyanine green angiography showed multiple patchy hypofluorescent lesions in the posterior pole. Optical coherence tomography confirmed choroidal thickening and disk swelling, and ultrasound biomicroscopy demonstrated bilateral serous ciliary body detachment. After excluding other systemic and ocular etiologies, a diagnosis of bilateral UC-associated panuveitis was made. The patient received topical steroids, retrobulbar injection of triamcinolone acetonide in the left eye and oral prednisone initiated at 60 mg/day and tapered gradually. Her BCVA improved to 20/20 in the right eye and 20/30 in the left eye after 9 months follow-up.

**Conclusion:**

UC-associated uveitis can manifest as bilateral panuveitis with ciliary body and choroidal detachment. Prompt diagnosis and immediate initiation of systemic corticosteroid therapy are critical for achieving optimal anatomical restoration and visual prognosis.

## Introduction

Inflammatory bowel disease (IBD), including ulcerative colitis (UC) and Crohn’s disease (CD), is a chronic relapsing inflammatory disorder primarily affecting the intestine and frequently associated with extraintestinal manifestations (EIMs) (1). Ocular extraintestinal manifestations (O-EIMs) are among the relatively frequent EIMs of IBD, ranking third after joint and skin involvement (2). Typical ocular symptoms include eye redness, ocular pain, photophobia, and blurred vision. Among inflammatory O-EIMs, uveitis is the most commonly reported manifestation, followed by episcleritis, scleritis, conjunctivitis, and optic neuritis (3).

Uveitis has been reported in 3.27% of patients with CD and 1.60% of those with UC, and the most common subtype is anterior uveitis, which primarily involves the anterior uveal structures (4). By contrast, inflammation involving the intermediate or posterior segment is classified as intermediate or posterior uveitis, respectively, whereas panuveitis refers to inflammation involving all segments without a single predominant site. Treatment of anterior uveitis often relies on topical steroids, whereas in intermediate, posterior, or panuveitis may require intravitreal or systemic steroids (3).

Timely diagnosis and early treatment of uveitis are essential to prevent vision loss. Given the limited description of uncommon forms of IBD-associated uveitis in the current literature, we report a rare case of UC-associated bilateral panuveitis with serous ciliary body detachment and choroidal detachment. Multimodal imaging facilitated the diagnosis, and timely corticosteroid treatment led to favorable anatomical and visual outcomes.

## Case report

A 51-year-old Asian woman presented to the ophthalmology clinic in September 2024 with a 10-day history of bilateral blurred vision, accompanied by ocular pain, redness, and photophobia. The symptoms had progressively worsened over the preceding week, and this was her first ophthalmic evaluation for the condition.

Four years earlier, she had been diagnosed with mildly active UC based on colonoscopic findings following incidental bowel wall thickening noted on abdominal CT. She was initially treated with oral mesalazine 4 g/day, which was later tapered to 1 g/day following mucosal healing. However, recurrent ulceration necessitated dose increase. One year prior to ocular symptom onset, oral mesalazine was switched to rectal suppositories due to newly identified right kidney atrophy and impaired renal function, which were suspected to be related to long-term mesalazine use. She had no history of skin or joint EIMs, and no concurrent UC flare was documented at the time of ocular presentation.

### Ophthalmic examination and multimodal imaging findings

At presentation, her best-corrected visual acuity (BCVA) was 20/60 in the right eye and 20/100 in the left eye, with normal intraocular pressure in both eyes. Slit-lamp examination revealed conjunctival hyperemia, fine pigmented keratic precipitates, moderate anterior chamber flare and cells, posterior synechiae, and pigment deposition on the anterior lens capsule.

**FIGURE 1:**
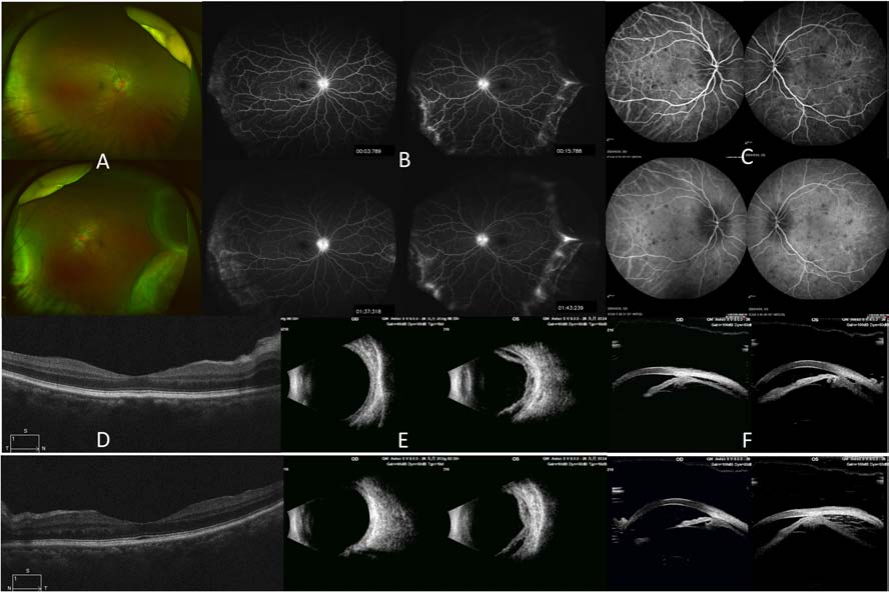
Multimodal imaging findings at presentation. **(A)** Fundus photograph showing bilateral optic disk edema and peripheral choroidal detachment in OS. **(B)** Fundus fluorescein angiography (FA) demonstrating bilateral disk hyperfluorescence and peripheral leakage. **(C)** Indocyanine green angiography (ICGA) showing multiple patchy hypofluorescent lesions scattered in the posterior pole from early to late phases. **(D)** Optical coherence tomography (OCT) revealing choroidal thickening and optic disk swelling in both eyes. **(E)** B-scan ultrasonography of OS confirming extensive peripheral choroidal detachment. **(F)** Ultrasound biomicroscopy demonstrating 360° ciliary body detachment in both eyes, more pronounced in OS.

Fundus examination showed bilateral optic disk edema and peripheral choroidal detachment in left eye (Figure 1A). Fundus fluorescein angiography demonstrated early bilateral optic disk hyperfluorescence with late leakage and mild peripheral vascular leakage (Figure 1B). Indocyanine green angiography (ICGA) showed multiple patchy hypofluorescent lesions scattered throughout the posterior pole from early to late phases (Figure 1C). Optical coherence tomography revealed choroidal thickening and optic disk swelling in both eyes (Figure 1D). B-scan ultrasonography of the left eye confirmed extensive peripheral choroidal detachment (Figure 1E). Ultrasound biomicroscopy (UBM) demonstrated 360° ciliary body detachment in both eyes, more prominent in the left eye (Figure 1F).

### Diagnostic impression and differential work-up

Based on the combined anterior, intermediate, and posterior segment involvement and her history of UC, bilateral panuveitis associated with UC was considered. Topical steroids eye drops were initiated in both eyes, and given the more severe involvement of the left eye, a retrobulbar injection of triamcinolone acetonide (40 mg) was administered to the left eye.

To exclude other possible causes of uveitis, an extensive diagnostic work-up was performed, including testing for syphilis, T-SPOT.TB, erythrocyte sedimentation rate, C-reactive protein, angiotensin-converting enzyme, antinuclear antibodies, antineutrophil cytoplasmic antibodies, anti-β2-glycoprotein antibodies, anti-cyclic citrullinated peptide antibodies, anti-perinuclear factor, anti-keratin antibodies, anti-mutated citrullinated vimentin antibodies, glucose-6-phosphate isomerase, dilute Russell’s viper venom time, anticardiolipin antibodies, HLA-B27, and HLA-B*51. All test results were negative or unremarkable.

### Therapeutic intervention and follow-up

**FIGURE 2:**
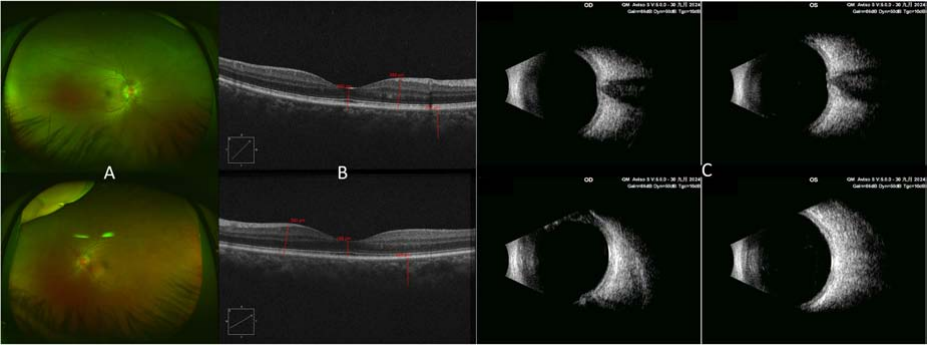
Early ocular improvement after initial anti-inflammatory treatment. Findings at the 1-week follow-up: **(A)** fundus photograph; **(B)** optical coherence tomography (OCT). **(C)** B-scan ultrasonography.

Topical corticosteroid eye drops were continued during follow-up. At the 1-week follow-up, anterior segment inflammation had improved, and both choroidal and ciliary body detachments were alleviated (Figure 2). However, her BCVA remained unchanged, and optic disk edema persisted. Given the persistent posterior segment involvement, oral prednisone was then started at 60 mg/day, together with gastric protection and calcium/vitamin D supplementation.

**FIGURE 3:**
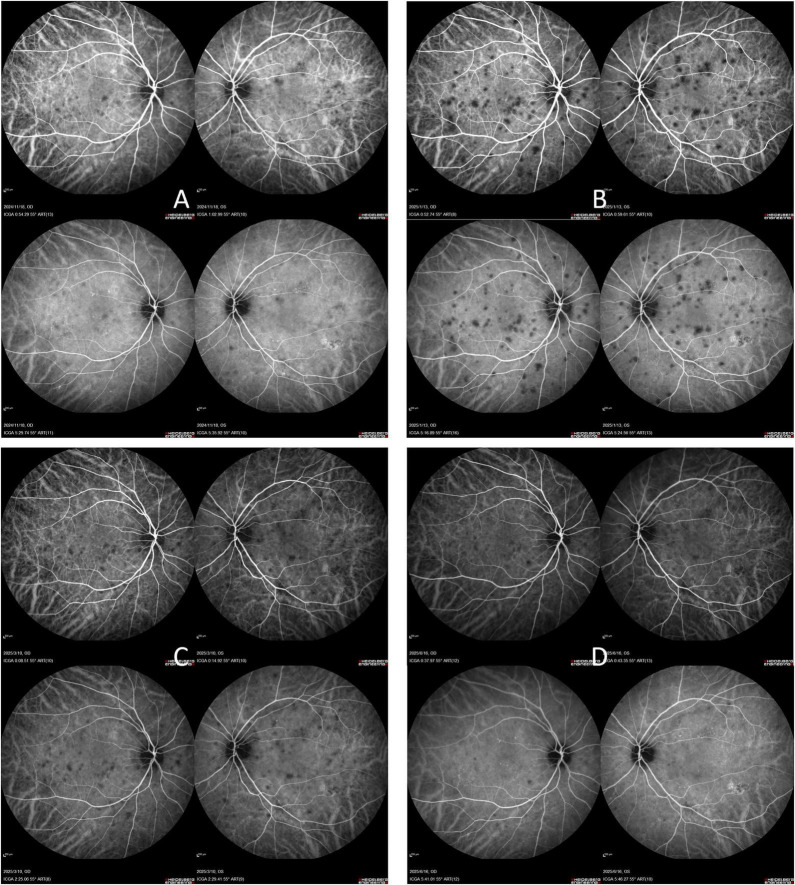
Serial indocyanine green angiography (ICGA) changes during follow-up. ICGA images obtained at **(A)** 2 months; **(B)** 4 months; **(C)** 6 months; **(D)** 9 months show dynamic changes in posterior pole hypofluorescent lesions.

After 1 month, her BCVA improved to 20/40 in the right eye and 20/60 in the left eye, with partial resolution of optic disk edema. Prednisone was then tapered by 5 mg per 2 weeks. At the 4-month follow-up, her BCVA further improved to 20/25 in the right eye and 20/40 in the left eye, with complete resolution of optic disk edema. However, ICGA revealed an increased number of hypofluorescent lesions (Figure 3). The use of anti-tumor necrosis factor (TNF)-α monoclonal antibody therapy was recommended, but the patient declined due to concerns about potential side effects. Prednisone was continued and tapered to a maintenance dose of 10 mg/day.

At the final follow-up in June 2025, BCVA had reached 20/20 OD and 20/30 OS, and ICGA demonstrated a reduction in hypofluorescent lesions compared to prior imaging. The patient remained on maintenance therapy with 2.5 mg/day of prednisone. The patient’s visual and anatomical outcomes were favorable, and no long-term complications were observed.

### Patient perspective

The patient reported that the initial visual loss and ocular discomfort significantly affected her daily activities. She had not realized that these ocular symptoms could be related to UC. After treatment, she noticed clear improvement in both symptoms and vision and felt reassured by the recovery.

## Discussion

The mechanisms underlying ocular extraintestinal manifestations in IBD remain incompletely understood, with proposed mechanisms including immune dysregulation, shared antigens between the gut and the eye, genetic predisposition, and cytokine-driven systemic inflammation (5, 6). Several risk factors for ocular involvement have also been reported, including genetic susceptibility and the presence of other extraintestinal manifestations, particularly articular involvement (2, 7, 8). These findings suggest that ocular inflammation in IBD is part of a broader systemic inflammatory process rather than an isolated ocular disorder.

Inflammatory bowel disease-associated panuveitis is rare and has been only infrequently reported, with few cases described in detail in the literature (9). To our knowledge, this is the first well-documented case report of IBD-associated uveitis with both ciliary body detachment and serous choroidal detachment. Notably, unlike choroidal detachment, which can be directly observed on fundus examination, ciliary body detachment may be more occult, particularly in the absence of hypotony. In such cases, UBM may be especially valuable for detecting ciliary body involvement.

The coexistence of choroidal and ciliary body detachment in this case may reflect severe intraocular inflammation with increased vascular permeability of the uveal tract, leading to exudative fluid accumulation. Similar mechanisms have been proposed in other autoimmune diseases, such as Vogt–Koyanagi–Harada disease and systemic lupus erythematosus, where immune complex deposition and choroidal vasculitis are considered key pathological features (10, 11). Notably, in our case, both choroidal and ciliary body detachments resolved rapidly following treatment with topical steroids and a retrobulbar injection of triamcinolone acetonide, further supporting the notion that these structural changes were inflammatory in nature and reversible.

Unlike episcleritis, which often parallels intestinal inflammation, uveitis is less closely associated with IBD activity (12). Previous studies have shown that uveitis is more frequent in patients with active CD than in those with inactive CD, whereas this pattern has not been observed in UC (13). Similarly, at the time of ocular symptom onset, our patient’s UC was clinically quiescent, with no concurrent gastrointestinal flare. These findings suggest that uveitis in IBD, particularly in UC, may follow a course distinct from intestinal symptoms, which may delay recognition of its association with the underlying bowel disease.

A key diagnostic point in this case was the exclusion of alternative causes of uveitis before confirming its association with UC. In clinical practice, infectious causes of uveitis, such as viral infection, syphilis, and tuberculosis, should be carefully excluded, and the differential diagnosis should also include non-infectious inflammatory disorders such as sarcoidosis, systemic lupus erythematosus, HLA-B27-associated disease, and Vogt–Koyanagi–Harada disease.

For the treatment of IBD-associated uveitis, anterior uveitis may respond to topical corticosteroids, whereas intermediate, posterior, and panuveitis often require more intensive therapy, including local steroid injection or systemic corticosteroids (3). For recurrent, severe, or steroid-dependent cases, steroid-sparing agents such as methotrexate, azathioprine, or mycophenolate mofetil may also be considered (2, 14). Among biologic therapies, anti–TNF-α agents, particularly adalimumab, have been shown to be effective in controlling intraocular inflammation and represent an important option for refractory disease (14, 15). In the present case, the persistence of hypofluorescent lesions on ICGA during corticosteroid therapy suggested suboptimal control of posterior pole inflammation. We therefore speculate that earlier initiation of adalimumab might have resulted in more rapid and complete control of ocular inflammation, although this remains to be validated.

This case underscores the need for multidisciplinary management in IBD-associated uveitis. Ophthalmologists are crucial for recognizing the full extent of ocular inflammation and monitoring treatment response, whereas gastroenterologists are needed to assess intestinal disease status and guide systemic therapy. In severe or refractory cases, rheumatology input may be valuable when considering corticosteroid-sparing or biologic treatment, and primary care physicians can assist with longitudinal monitoring of systemic adverse effects and treatment adherence. Such interdisciplinary collaboration is essential for timely diagnosis, appropriate escalation of therapy, and coordinated long-term care.

## Conclusion

This case demonstrates that UC-associated uveitis may present as bilateral panuveitis with uncommon structural manifestations, including serous ciliary body and choroidal detachment. Recognition of these atypical features, supported by multimodal imaging, is essential for timely diagnosis. Early topical steroid treatment may lead to rapid anatomical improvement, while persistent posterior segment inflammation may require prolonged systemic steroids or biologic therapy. Greater awareness of such presentations may help ophthalmologists and gastroenterologists optimize multidisciplinary management of ocular involvement in IBD.

## Data Availability

The original contributions presented in this study are included in the article/supplementary materials, further inquiries can be directed to the corresponding author.
